# Association between body roundness index and trajectories of depressive symptoms among Chinese middle-aged and older adults: a nationwide cohort study from CHARLS

**DOI:** 10.1186/s40359-025-03621-z

**Published:** 2025-11-18

**Authors:** Dehua Zhao, Xiaoqing Long, Jisheng Wang

**Affiliations:** Department of Clinical Pharmacy, The Third Hospital of Mianyang (Sichuan Mental Health Center), Mianyang, China

**Keywords:** BRI, Depressive symptoms, Trajectories, GBTM, CHARLS

## Abstract

**Background:**

The relationship between body roundness index (BRI) and longitudinal trajectories of depressive symptoms remains underexplored in middle-aged and older Chinese populations. We therefore aimed to investigate this association utilizing data from a nationally representative longitudinal study.

**Methods:**

This study utilized longitudinal data from the China Health and Retirement Longitudinal Study (CHARLS) spanning 2011 to 2020. Depressive symptoms were assessed using the validated 10-item Center for Epidemiologic Studies Depression Scale (CESD-10). To capture the heterogeneity in symptom progression, we employed group-based trajectory modeling (GBTM). The association between BRI and depressive symptom trajectories was subsequently examined through multivariable logistic regression analyses, complemented by restricted cubic spline (RCS) regression and stratified analyses to assess potential non-linear relationships and subgroup variations.

**Results:**

The analysis included 10,335 CHARLS participants with repeated depressive symptom assessments from 2011 to 2020. GBTM identified two distinct trajectories: stable low depressive symptoms (51.80% of participants) and persistent high depressive symptoms (48.20%). After adjusting for potential confounders, multivariable logistic regression revealed an inverse association between BRI and persistent high depressive symptoms (OR = 0.96, 95% CI: 0.93–0.99, *P* = 0.019). RCS analysis confirmed a linear inverse relationship (*P* for non-linearity = 0.116). These inverse associations persisted across stratified analyses with no significant interaction effects between subgroups (*P*-interaction > 0.05).

**Conclusion:**

These findings indicated that elevated BRI values were associated with more favorable depressive symptom trajectories among Chinese middle-aged and older adults.

**Supplementary Information:**

The online version contains supplementary material available at 10.1186/s40359-025-03621-z.

## Introduction

Depression constitutes a major global public health challenge due to its high prevalence and significant contribution to disability and mortality burdens. According to the World Health Organization estimates, approximately 350 million people worldwide experience depression, with incidence rates rising annually [1]. Elderly populations face elevated depression risk due to physiological decline, social role transitions, and higher comorbidity prevalence [2]. Results from a national survey in China revealed that depressive symptoms affect nearly 43% of women and 30% of men aged 45 years and above [3]. Depression manifests through core symptoms of persistent depressed mood, cognitive impairment, somatic disturbances, and diminished social functioning [4]. Severe cases may involve suicidal ideation requiring crisis intervention [5]. The etiology integrates genetic predisposition, neurobiological alterations, and psychosocial determinants [6, 7]. Significant clinical heterogeneity exists across symptom profiles, disease trajectories, treatment responsiveness, and prognostic outcomes, complicating therapeutic management [8]. These challenges necessitate enhanced prevention strategies and targeted interventions to mitigate disease burden.

Emerging evidence indicates that key obesity indices—including body roundness index (BRI), waist circumference (WC), and body mass index (BMI)—exhibit significant associations with depressive symptoms [9–11]. BMI and WC represent the most widely employed obesity indicators in epidemiological research. Both metrics feature prominently in studies examining the association between obesity and depressive symptoms. In a cohort study [11], males with obesity (BMI-defined) were less likely to suffer from depressive symptoms than males with a normal weight (HR = 0.51, 95% CI = 0.35–0.74). Similarly, males with abdominal obesity (WC-defined) showed reduced depression likelihood compared to those without abdominal obesity (HR = 0.78, 95% CI = 0.64–0.93). However, BMI constitutes a crude proxy for adiposity that fails to differentiate lean mass from fat mass [12]. Similarly, WC inadequately characterizes body morphology, and adiposity percentage [13]. BRI represents a novel anthropometric index combining WC and height, offering enhanced assessment of abdominal adiposity and individualized body composition [14]. Unlike BMI, the BRI quantifies abdominal adiposity by integrating WC-height relationships to estimate visceral fat accumulation relative to overall body proportion. Furthermore, BRI provides superior quantification of visceral fat distribution compared to WC alone. In a large-scale cross-sectional analysis of 26,332 participants [15], the BRI demonstrated superior diagnostic performance for identifying metabolically obese normal-weight individuals among 12 anthropometric indicators, with significantly greater accuracy than both BMI and WC. Consequently, BRI exhibits superior ability to characterize obesity severity compared to BMI and WC [14]. However, research examining the association between BRI and depressive symptoms yields inconsistent findings, with some studies reporting that elevated BRI levels increase the risk of depressive symptoms while others indicate protective associations [9, 10]. This inconsistency may stem from differences in population characteristics, such as age, race, and cultural background, as well as variations in confounding factors and the use of different indices and criteria for measuring depressive symptoms [9, 10].

Most studies examining the relationships of BRI and depressive symptoms have relied on a specific point assessment rather than longitudinal symptom progression [9, 10]. This approach inadequately captures clinical heterogeneity. In contrast, group-based trajectory modeling (GBTM) simultaneously estimates multiple distinct trajectories through finite mixture modeling [16]. This method identifies subpopulations with differential depressive symptom courses, thereby capturing heterogeneity in longitudinal patterns and providing comprehensive insight into symptom evolution [17]. Consequently, this study aimed to investigate the association between BRI and depressive symptom trajectories using repeated measurements of depressive symptoms in a nationally representative sample of Chinese middle-aged and older adults, aiming to provide insights for developing targeted mental health policies and interventions for aging populations.

## Methods

### Study participants

This study utilized data from five waves (2011, 2013, 2015, 2018, and 2020) of the China Health and Retirement Longitudinal Study (CHARLS). CHARLS employs a nationally representative cohort design, with its 2011 baseline survey implementing a multistage probability-proportional-to-size (PPS) sampling strategy. The survey encompasses 28 provinces, 150 counties/districts, and 450 villages/communities across China; subsequent waves (2013, 2015, 2018, and 2020) collected longitudinal data on health status and determinants among middle - aged and older Chinese adults.

Ethical approval for this study was granted by the Biomedical Ethics Review Committee of Peking University (Approval ID: IRB00001052-11015). The research complies with institutional and national ethical guidelines, including the principles outlined in the Declaration of Helsinki (1964) and its subsequent amendments. Our study included participants aged ≥ 45 years with baseline BRI measurements and ≥ 3 longitudinal depressive symptom assessments.

### Assessment of BRI

BRI quantifies body shape using WC and height measurements. Trained personnel obtained baseline anthropometric data through standardized protocols: WC was assessed at the umbilical level using a non-stretch tape measure at the end of normal expiration. Height was measured with a Seca 213 stadiometer (Seca GmbH, Hamburg, Germany), with participants positioned barefoot in the standing position on the instrument’s baseplate. WC and height were measured to the nearest 0.1 cm. The BRI is calculated via the formula proposed by Thomas et al. [18] The equation is as follows:

$\:BRI=364.2-365.5\times\:\sqrt{1-\left(\frac{(\frac{WC}{2\pi\:})^2}{(0.5\times\:H)^2}\right)}$, where WC represents waist circumference (cm) and H represents height (cm).

### Assessment of depressive symptoms

The Center for Epidemiologic Studies Depression Scale (CESD-10) was utilized to evaluate participants’ depressive symptoms [7, 19]. This 10-item scale offers four response options for each item, scored from 0 to 3 points: 0 indicates symptoms present rarely or never (< 1 day), 1 suggests symptoms sometimes or sporadically (1–2 days), 2 reflects symptoms occurring a moderate amount of the time (3–4 days), and 3 signifies frequent or constant symptoms (5–7 days). Items five and eight are reverse-scored. Total scores range from 0 to 30, with higher scores indicating a greater likelihood of major depressive disorder. A cutoff score of ≥ 10 was employed to identify participants with significant depressive symptoms [20, 21].

### Assessment of covariates

A cohort study revealed that both socioeconomic status and social activities were associated with depressive symptoms in adults aged 50 years [7]. Cross-sectional analyses of the 2020 CHARLS showed that inactive weekly physical activity was associated with an increased risk of depression [22]. Ju C et al. [23] demonstrated that current smoking and alcohol consumption may confer modest protective effects against depressive symptoms. Furthermore, a cohort study suggested that increased C-reactive protein (CRP) predicted more depressive symptoms in participants with higher social strain [24]. In a cross-sectional study [25], older individuals, especially women, were more likely to suffer from depression. Zhao L et al. [26] showed that gender and marital status had significant effects on depression among Chinese middle-aged and older people. Moreover, a prospective study indicated that individuals with chronic diseases or multimorbidity were more likely to be depressed [27]. Informed by this collective evidence and clinical consensus, we comprehensively adjusted for sociodemographic characteristics, behavioral factors, health status indicators, and chronic conditions. Specifically, sociodemographic characteristics included age, gender, residence (rural or urban), education level (elementary school or below, middle school, high school, and college or above), marital status (married or not married), health insurance (urban employee medical insurance, urban and rural resident medical insurance, other medical insurance, or no insurance), and household consumption. Behavioral factors included social activities, physical activities (vigorous, moderate, or other), smoking status (current, former, or never smokers), drinking status (current, former, or never drinkers). Health status indicators included self rated health (good, fair, or poor) and CRP. Chronic diseases included hypertension, dyslipidemia, diabetes, cancer or malignant tumor, chronic lung diseases, liver diseases, heart diseases, stroke, kidney diseases, stomach or other digestive diseases, memory related diseases, arthritis or rheumatism, and asthma.

### Statistical analysis

Covariate data were missing for 3,032 participants (29.34%). Under the assumption that these data were missing at random, we applied multiple imputation by chained equations (MICE) to generate five multiply imputed datasets, thereby preserving statistical power while mitigating selection bias. The GBTM was employed to identified depressive symptom trajectories over time. Models specifying 2–6 trajectory classes were evaluated sequentially. We simultaneously evaluated alternative growth parameters (linear, quadratic, or cubic) for each trajectory to determine the optimal polynomial order describing depressive symptom progression. Selection of the optimal model employed the Bayesian Information Criterion (BIC), Akaike’s Information Criterion (AIC), average posterior probabilities (AvePP), and class size criteria. The following thresholds were applied: AvePP > 0.70 for all classes, and minimum class size ≥ 5% of the sample. Models satisfying these requirements were then compared, with preference given to those exhibiting lower BIC and AIC values [28, 29].

Normally distributed continuous variables were reported as mean ± standard deviation (SD); non-normally distributed variables as median and interquartile range (IQR). Categorical variables were presented as frequencies (percentages). Group differences across depressive symptom trajectories were assessed using chi-square tests for categorical variables, one-way ANOVA for normally distributed continuous variables, and Kruskal-Wallis tests for non-normally distributed variables.

The odds ratios (ORs) and 95% confidence intervals (CIs) were estimated to investigate the relationship between BRI and depressive symptom trajectories using multivariable logistic regression models. We employed four models to incrementally adjust for covariates. Model 1 was unadjusted. Model 2 adjusted for sociodemographic characteristics. Model 3 incorporated further adjustment for behavioral factors and health indicators. Model 4 included additional adjustment for chronic diseases. The four models incorporating varying levels of covariate adjustment were frequently employed in prior research to assess the robustness of the observed associations [30–32]. In addition, the RCS analysis was employed to explore the potential nonlinear association between BRI and depressive symptom trajectories. The sociodemographic analysis revealed significant disparities (*P* < 0.05) between the two depressive symptom trajectories across multiple variables: age, gender, marital status, residence, education level, and health insurance. Notably, subgroup analyses for college or above education and other medical insurance categories exhibited limited statistical power due to insufficient sample sizes. Consequently, we selected age (< 65 vs. ≥65 years), gender, marital status, and residence for stratified analysis. Interaction effects were evaluated using likelihood ratio tests, with heterogeneity quantified through interaction *P*-values.

All analyses were conducted in R 4.3.0 (R Foundation). Statistical significance was defined as *P* < 0.05 for all tests.

## Results

### Estimated depressive symptom trajectories

**Fig. 1:**
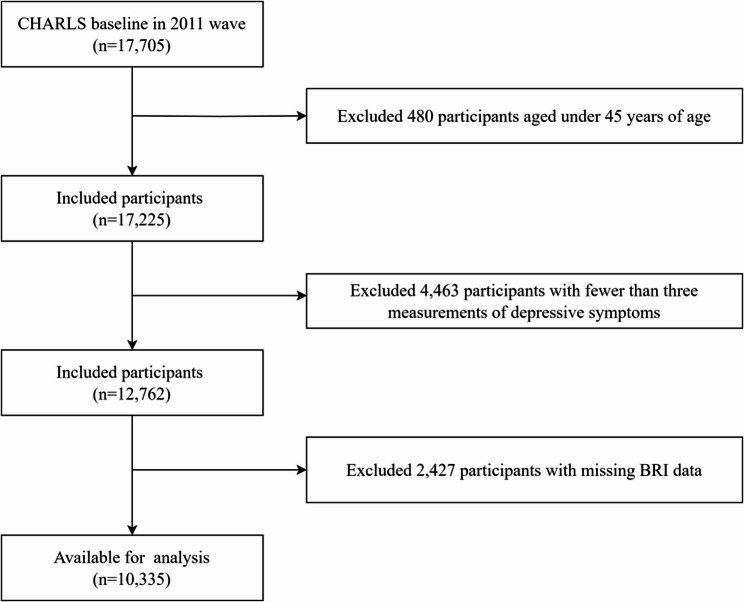
Flow chart of participants selection process

The 2011 baseline cohort comprised 17,705 participants. We excluded: (1) individuals aged < 45 years (*n* = 480), (2) those with < 3 depressive symptom assessments (*n* = 4,463), and (3) participants missing BRI data (*n* = 2,427). After exclusions, 10,335 participants with ≥ 3 longitudinal depressive symptom assessments were included in the final analysis. Fig. 1 detailed the selection process.

**Fig. 2:**
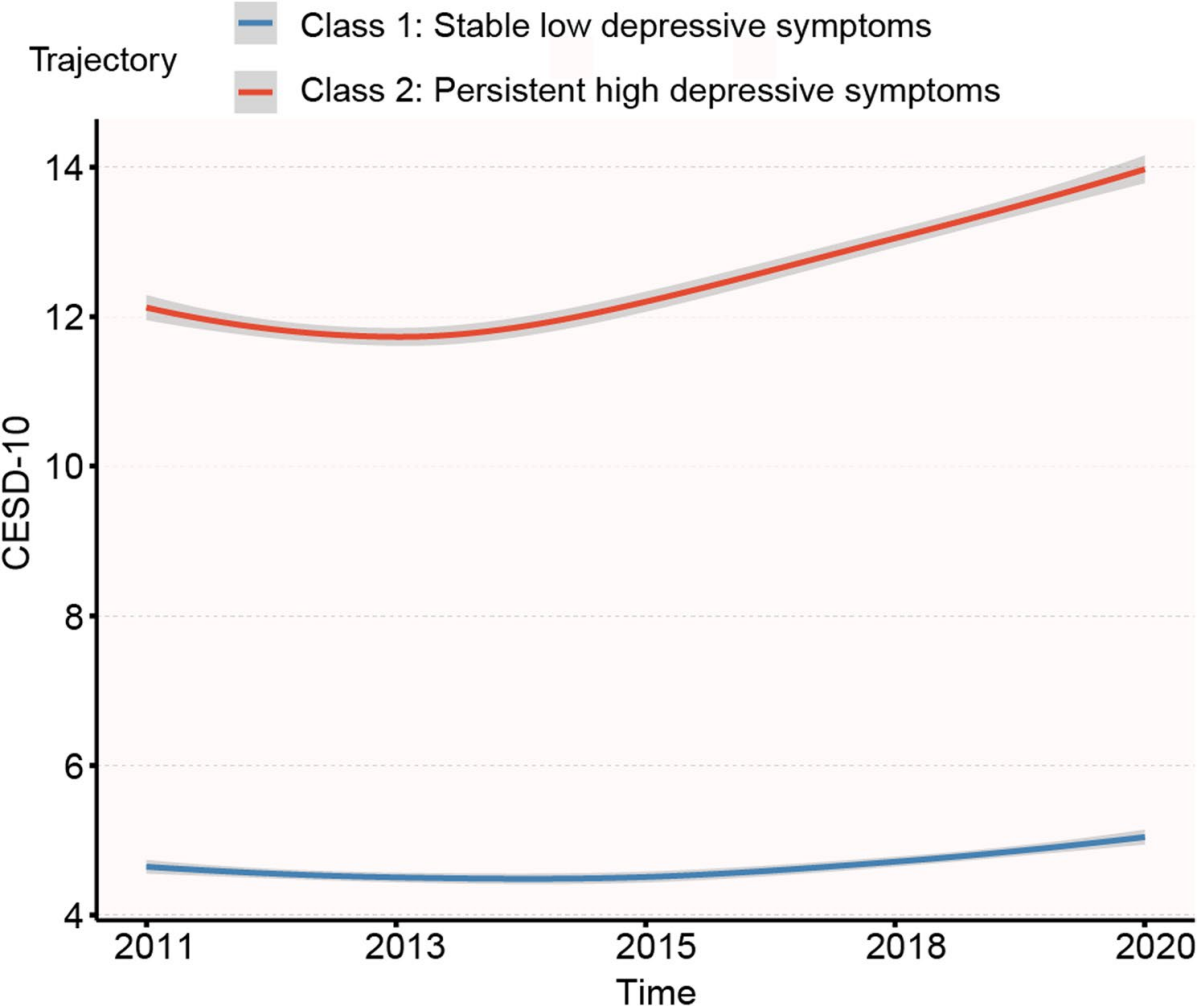
Trajectories of depressive symptoms

Trajectory models ranging from 2 to 6 classes were used to explain the heterogeneity in dynamic change of depressive symptoms during the study period. Although higher-order trajectory models (3–6 classes) were associated with further improvement in BIC and AIC, these models yielded some trajectories with AvePP < 0.70 (Supplementary Table 1). Specifically, for the 3-trajectory model, AvePP = 0.69 (Class 3); for the 4-trajectory model, 0.45 (Class 2) and 0.63 (Class 4); for the 5-trajectory model, 0.54 (Class 2), 0.65 (Class 3), and 0.58 (Class 5); and for the 6-trajectory model, 0.45 (Class 2), 0.52 (Class 3), 0.58 (Class 4), and 0.47 (Class 6). Consequently, according to the selection criteria of the GBTM model, we selected the 2- group depressive symptoms trajectory model (1 linear and 1quadratic) for use in subsequent analyses. The parameter estimates for the 2-group trajectory model were presented in Table 1. The AvePP values of two trajectory groups were 0.85 and 0.91 with each group representing more than 5% of the total sample. The final trajectories were qualitatively labeled according to their distinct longitudinal patterns (Fig. 2): Class 1 represented a stable low depressive symptoms (*n* = 5,354, 51.86% of sample), while Class 2 exhibited persistent high depressive symptoms (*n* = 4,981, 48.20%).

**Table 1:** Parameter estimates of the 2-group trajectory model

Trajectory group	parameter	Intercept	coefficient	SE	Wald	*P*-value
Class 1	Linear	4.82	0.10	0.03	3.81	< 0.001
	Quadratic	4.91	0.02	0.01	4.00	< 0.001
Class 2	Linear	10.93	0.49	0.03	15.31	< 0.001
	Quadratic	11.12	0.14	0.01	18.14	< 0.001

*SE* Standard error

### Baseline characteristics

The depressive symptom scores alongside corresponding participant ages at each assessment wave were presented in Supplementary Table 2. The baseline characteristics of participants across distinct depressive symptom trajectory groups were delineated in Table 2. The final analytical sample comprised 10,335 participants (4,955 males and 5,380 females), with a mean age of 57.66 ± 8.41 years. The median annual household consumption was 16,280.00 (8,996.00, 27,420.00) yuan. Geographically, 81.98% of participants resided in rural areas. Educational attainment was predominantly low, with 67.06% having completed elementary school or less. Marital status data revealed that 89.78% of participants were married. Regarding healthcare coverage, 83.69% were enrolled in the urban and rural resident medical insurance. Notably, individuals within the “persistent high depressive symptoms” trajectory group exhibited a propensity to be female, to have advanced age, to possess lower educational attainment and household consumption levels, to reside in rural regions, to be unmarried, to lack health insurance, and to report poor self-rated health.

**Table 2:** Baseline characteristics of participants according to depressive symptom trajectories

Variables	Total (*n* = 10,335)	Stable low depressive symptoms (*n* = 5,354)	Persistent high depressive symptoms (*n* = 4,981)	*P*-value
Gender, n (%)				< 0.001
Male	4,955 (47.94)	3,053 (57.02)	1,902 (38.19)	
Female	5,380 (52.06)	2,301 (42.98)	3,079 (61.81)	
Residence, n (%)				< 0.001
Rural	8,473 (81.98)	4,100 (76.58)	4,373 (87.79)	
Urban	1,862 (18.02)	1,254 (23.42)	608 (12.21)	
Education level, n (%)				< 0.001
Elementary school or below	6,931 (67.06)	3,122 (58.31)	3,809 (76.47)	
Middle school	2,246 (21.73)	1,386 (25.89)	860 (17.27)	
High school	1,002 (9.70)	712 (13.3)	290 (5.82)	
College or above	156 (1.51)	134 (2.5)	22 (0.44)	
Marital status, n (%)				< 0.001
Married	9,279 (89.78)	4,934 (92.16)	4,345 (87.23)	
Not married	1,056 (10.22)	420 (7.84)	636 (12.77)	
Age (year), Mean ± SD	57.66 ± 8.41	57.36 ± 8.55	57.99 ± 8.25	< 0.001
CRP (mg/L), Median (IQR)	1.02 (0.54, 2.08)	1.03 (0.55, 2.07)	1.00 (0.54, 2.10)	0.838
BRI, Mean ± SD	4.23 ± 1.37	4.19 ± 1.35	4.26 ± 1.39	0.013
Health insurance, n (%)				< 0.001
Urban employee medical insurance	997 (9.65)	743 (13.88)	254 (5.1)	
Urban and rural resident medical insurance	8,649 (83.69)	4,258 (79.53)	4,391 (88.15)	
Other medical insurance	110 (1.06)	77 (1.44)	33 (0.66)	
No insurance	579 (5.60)	276 (5.16)	303 (6.08)	
Household consumption (yuan), Median (IQR)	16,280.00 (8,996.00, 27,420.00)	17,840.00 (9,680.00, 29,860.00)	14,780.00 (8,380.00, 25,060.00)	< 0.001
Hypertension, n (%)				0.052
No	6,370 (61.64)	3,348 (62.53)	3,022 (60.67)	
Yes	3,965 (38.36)	2,006 (37.47)	1,959 (39.33)	
Dyslipidemia, n (%)				0.361
No	7,690 (74.41)	4,004 (74.79)	3,686 (74)	
Yes	2,645 (25.59)	1,350 (25.21)	1,295 (26)	
Diabetes, n (%)				< 0.001
No	9,579 (92.69)	5,008 (93.54)	4,571 (91.77)	
Yes	756 (7.31)	346 (6.46)	410 (8.23)	
Cancer or malignant tumor, n (%)				0.025
No	10,230 (98.98)	5,311 (99.2)	4,919 (98.76)	
Yes	105 (1.02)	43 (0.8)	62 (1.24)	
Chronic lung diseases, n (%)				< 0.001
No	9,339 (90.36)	4,974 (92.9)	4,365 (87.63)	
Yes	996 (9.64)	380 (7.1)	616 (12.37)	
Liver diseases, n (%)				< 0.001
No	9,926 (96.04)	5,179 (96.73)	4,747 (95.3)	
Yes	409 (3.96)	175 (3.27)	234 (4.7)	
Heart diseases, n (%)				< 0.001
No	9,185 (88.87)	4,874 (91.03)	4,311 (86.55)	
Yes	1,150 (11.13)	480 (8.97)	670 (13.45)	
Stroke, n (%)				< 0.001
No	10,155 (98.26)	5,287 (98.75)	4,868 (97.73)	
Yes	180 (1.74)	67 (1.25)	113 (2.27)	
Kidney diseases, n (%)				< 0.001
No	9,673 (93.59)	5,121 (95.65)	4,552 (91.39)	
Yes	662 (6.41)	233 (4.35)	429 (8.61)	
Stomach or other digestive diseases, n (%)				< 0.001
No	7,896 (76.40)	4,405 (82.27)	3,491 (70.09)	
Yes	2,439 (23.60)	949 (17.73)	1,490 (29.91)	
Memory related diseases, n (%)				< 0.001
No	10,235 (99.03)	5,319 (99.35)	4,916 (98.7)	
Yes	100 (0.97)	35 (0.65)	65 (1.3)	
Arthritis or rheumatism, n (%)				< 0.001
No	6,719 (65.01)	3,957 (73.91)	2,762 (55.45)	
Yes	3,616 (34.99)	1,397 (26.09)	2,219 (44.55)	
Asthma, n (%)				< 0.001
No	9,990 (96.66)	5,230 (97.68)	4,760 (95.56)	
Yes	345 (3.34)	124 (2.32)	221 (4.44)	
Physical activities, n (%)				0.034
Vigorous activities	1,722 (16.66)	843 (15.75)	879 (17.65)	
Moderate activities	1,375 (13.30)	716 (13.37)	659 (13.23)	
Other activities	7,238 (70.03)	3,795 (70.88)	3,443 (69.12)	
Social activities, n (%)				< 0.001
No	5,039 (48.76)	2,388 (44.6)	2,651 (53.22)	
Yes	5,296 (51.24)	2,966 (55.4)	2,330 (46.78)	
Smoking status, n (%)				< 0.001
Current smokers	3,247 (31.42)	1,858 (34.7)	1,389 (27.89)	
Former smokers	855 (8.27)	520 (9.71)	335 (6.73)	
Never smokers	6,233 (60.31)	2,976 (55.58)	3,257 (65.39)	
Drinking status, n (%)				< 0.001
Current drinkers	2,660 (25.74)	1,641 (30.65)	1,019 (20.46)	
Former drinkers	818 (7.91)	463 (8.65)	355 (7.13)	
Never drinkers	6,857 (66.35)	3,250 (60.7)	3,607 (72.42)	
Self-rated health, n (%)				< 0.001
Good	2,427 (23.48)	1,757 (32.82)	670 (13.45)	
Fair	5,126 (49.60)	2,846 (53.16)	2,280 (45.77)	
Poor	2,782 (26.92)	751 (14.03)	2,031 (40.77)	

*CRP* C-reactive protein, *BRI* Body roundness index, *SD* Standard deviation, *IQR* Interquartile range

###  Association between BRI and depressive symptom trajectories

**Fig. 3:**
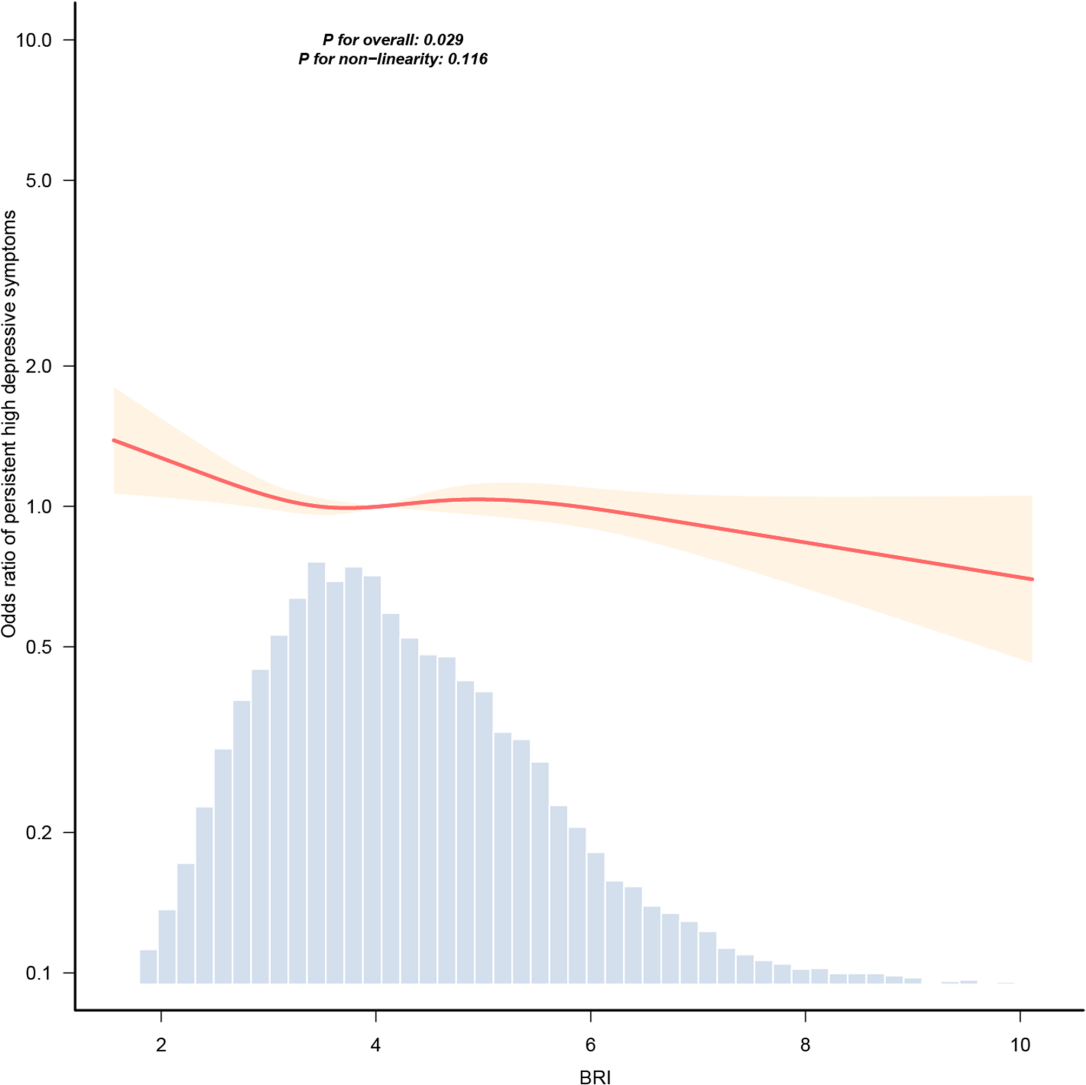
Dose-response relationship between BRI and persistent high depressive symptoms. Adjustment factors included sociodemographic characteristics, behavioral factors, health indicators, and chronic diseases

The association between BRI and depressive symptom trajectories was presented in Table 3. In unadjusted model, BRI demonstrated a positive association with persistent high depressive symptoms. However, the fully adjusted model revealed a statistically significant inverse association between BRI and persistent high depressive symptoms (OR = 0.96, 95% CI: 0.93–0.99, *P* = 0.019). This protective relationship demonstrated robustness across successive models incorporating different covariate adjustments (Models 2–3). The coefficients for all covariates were included in Supplementary Table 3. Similarly, the RCS analysis demonstrated a linear dose-response relationship between BRI levels and persistent high depressive symptoms (*P* for non-linearity = 0.116) (Fig. 3), suggesting a consistent inverse association across the exposure range.

**Table 3:** Association between BRI and depressive symptom trajectories

Models	OR (95%CI)	*P*-value
Model 1	1.04 (1.01–1.07)	0.013
Model 2	0.95 (0.93–0.99)	0.004
Model 3	0.96 (0.93–0.99)	0.017
Model 4	0.96 (0.93–0.99)	0.019

*CI* Confidence interval, *OR* Odds ratio

Model 1 was unadjusted; model 2 was adjusted for sociodemographic characteristics; model 3 was further adjusted for behavioral factors and health indicators; and model 4 was additionally adjusted for chronic diseases

### Stratified analyses

**Fig. 4:**
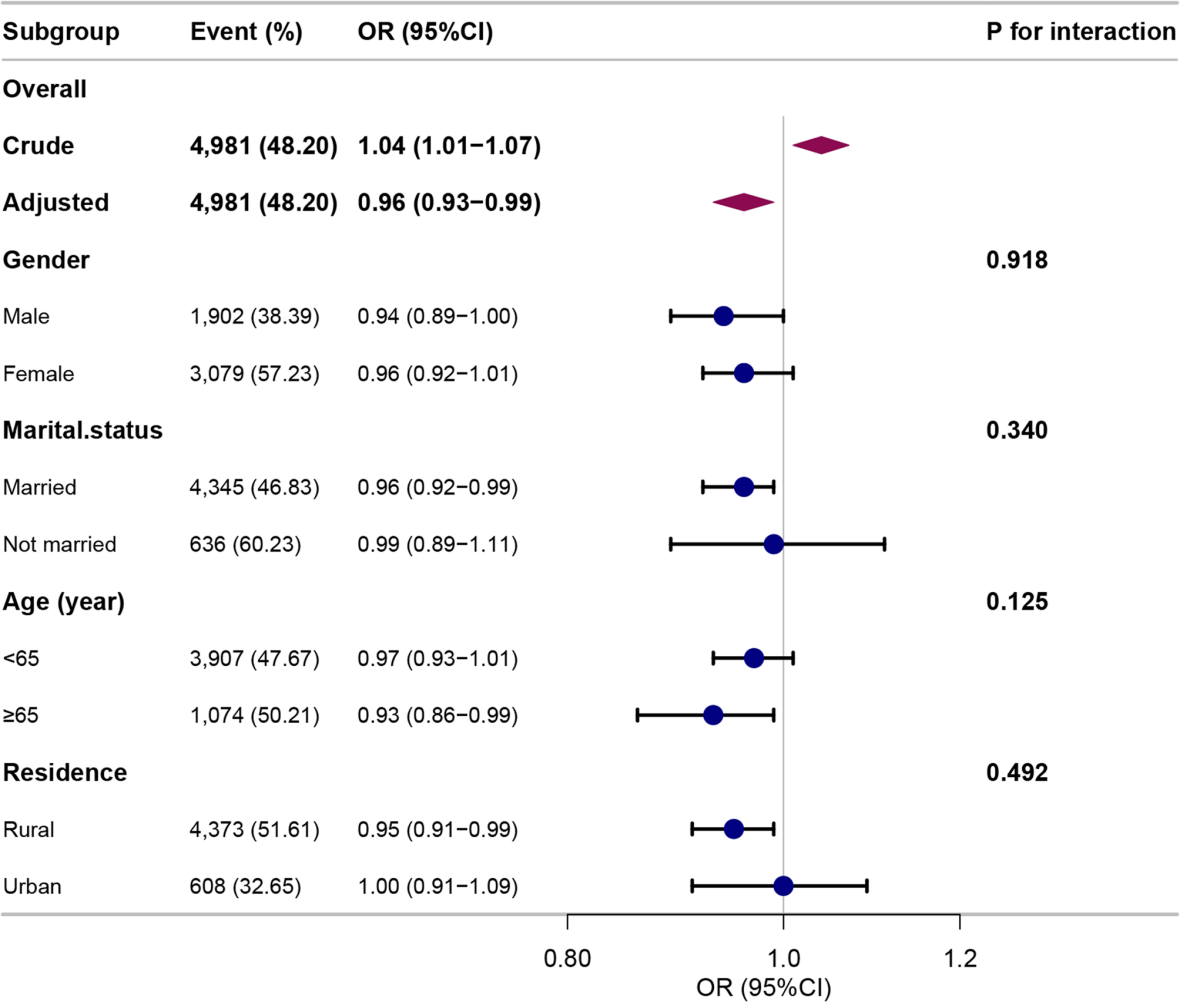
Stratified analyses of the association between BRI and depressive symptom trajectories. Stratified models were adjusted for sociodemographic characteristics, behavioral factors, health indicators, and chronic diseases, with the stratification variable excluded from adjustments

In stratified analyses (Fig. 4), the protective association between higher BRI and reduced risk of persistent high depressive symptoms appeared more pronounced among males, married participants, older adults (≥ 65 years), and rural residents. However, no statistically significant interaction effects were observed in any subgroup (*P* > 0.05).

## Discussion

This longitudinal study identified two distinct depressive symptom trajectory patterns among 10,335 middle-aged and older Chinese adults, derived from five assessment waves (2011–2020). Our study found that higher BRI values were associated with a reduced likelihood of developing persistent high depressive symptoms in a dose–response pattern. Stratified analyses demonstrated this inverse association across different sociodemographic characteristics, thus positioning BRI as a valuable predictor for evaluating the risk of depressive symptoms in this population.

Our findings were consistent with existing evidence on body composition-mental health associations [10, 33]. A cross-sectional analysis of 2013 CHARLS data revealed an inverse association between obesity and depressive symptoms among Chinese adults, with adjusted OR of 0.74 (95% CI: 0.57–0.97) in men and 0.73 (95% CI: 0.61–0.88) in women [10]. Similar inverse associations emerged in another cross-sectional study of 11,842 Chinese adults aged ≥ 65 years [33]. In the fully adjusted model, participants in the highest BRI quartile (Q4) showed significantly lower depressive symptom odds compared to the lowest quartile (Q1) (OR = 0.72, 95% CI: 0.62–0.82, *P* < 0.001) [33]. Additionally, a cohort study provided evidence for an inverse relationship between BRI and depressive symptoms in both females (OR = 0.91, 95% CI: 0.84–0.98) and males (OR = 0.88, 95% CI: 0.79–0.98), offering support for the “jolly fat” hypothesis among Chinese middle-aged and older adults [34]. However, these prior studies assessed depressive symptoms at a single timepoint using dichotomization thresholds lacking consensus (e.g., CESD-10 cutoffs of > 10 or 12). As demonstrated by He D et al. [35], such approaches introduce diagnostic instability: approximately 15% of participants reclassified depression status when thresholds changed or measurements were repeated. Therefore, to address these methodological limitations, we employed GBTM. This approach enabled robust longitudinal analysis of the relationship of BRI and depressive symptoms while capturing symptom dynamics beyond binary classifications.

Nevertheless, Some studies have reported findings that diverge from ours. In a cross-sectional study using data from the 2011–2018 NHANES [9], BRI was positively correlated with depression after adjusting for potential covariates. For each one-unit increase in BRI, the prevalence of depression increased by 8% (OR = 1.08, 95% CI: 1.05–1.10, *P* < 0.001) [9]. Furthermore, a cross-sectional study of 3,213 Iranian adults found no significant association between BRI and depression after multivariable adjustment (OR = 1.22, 95% CI: 0.95–1.57, *P* = 0.120) [36]. Several methodological and population-based factors may account for the discrepancies between our findings and those reported in aforementioned studies. First, the two studies focused on Iranian and American adults, whereas our research targeted middle-aged and older adults in China, populations that differ in demographic characteristics such as ethnicity and age. Second, the instruments used to assess depressive symptoms or depression varied across studies. Third, while the two cross-sectional designs captured point-in-time relationships, our longitudinal cohort study employed GBTM to systematically characterize depressive symptom evolution across multiple waves, providing greater statistical power to detect associations over time.

The observed inverse relationship between obesity and depression, commonly referred to as the “jolly fat” phenomenon, may be attributed to multiple neurobiological and sociocultural factors [37–42]. From a physiological perspective, dietary restriction, a common weight-loss strategy, has been identified as a potential depressive trigger [37]. Consequently, obese individuals who maintain unrestricted eating patterns may exhibit lower depression rates due to the absence of this dietary stressor [37]. At the molecular level, leptin signaling appears to play a crucial mediating role [38]. Leptin, an adipose-derived protein hormone, regulates energy homeostasis through hypothalamic receptor binding, suppressing appetite while enhancing energy expenditure [38]. Yang et al. [38] revealed that the leptin receptor protein expression was significantly reduced in comorbid depression-obesity rodent models. This receptor downregulation may contribute to weight gain in depressed subjects through disrupted metabolic signaling [38]. Neuropeptide Y (NPY), a 36-amino acid orexigenic neurotransmitter, provides another mechanistic explanation [39]. Beyond its appetite-stimulating properties, NPY administration has demonstrated antidepressant effects in the forced swim test (FST), a validated preclinical model for antidepressant screening [39, 40]. NPY-treated subjects exhibited increased swimming duration and reduced immobility-behavioral markers interpreted as reduced depressive-like symptoms [40]. Cultural influences further complicate this relationship, particularly in Chinese populations [41, 42]. Traditional Chinese cultural values associate midlife weight gain with prosperity and good fortune, potentially fostering more positive obesity perceptions that buffer against depressive symptoms [42]. This cultural lens offers an important sociocultural dimension to the “jolly fat” hypothesis that complements existing neurobiological explanations.

Beyond the “jolly fat” hypothesis, current evidence emphasizes substantial heterogeneity in depressive symptom manifestations [43–45]. Crucially, symptom profiles, neurobiological signatures, and inflammatory correlates exhibit significant variation across demographic strata, metabolic phenotypes, and depression subtypes [44, 45]. This heterogeneity framework explains why obesity-depression associations demonstrate critical variation across racial/ethnic, socioeconomic, gender, and age strata [9, 10, 33, 34, 36].

This study has several strengths. First, it uses high-quality longitudinal data from CHARLS, collected through the PPS random sampling method, which ensures national representativeness and enhanced external validity. Second, the application of GBTM to a nationally representative cohort over a 10-year period enables precise tracking of depressive symptom progression, surpassing the limitations of single-time-point assessments. Third, the use of RCS analysis helps to quantify the dose- response relationship between BRI and depressive symptom trajectories.

While this study possesses notable methodological strengths, several limitations warrant consideration. First, despite comprehensive adjustment for relevant covariates in our analytical models, the potential for residual confounding persists, which may influence the observed associations. Second, the generalizability of our findings may be constrained by the study’s exclusive focus on Chinese adults aged 45 years and older, necessitating cautious interpretation when considering other demographic groups. Third, our assessment of depressive symptoms relied on the CESD-10, a self-reported measure that may be susceptible to recall bias. Nevertheless, the CESD-10 has demonstrated excellent psychometric properties across multiple validation studies, with consistently high internal consistency (Cronbach’s α coefficients ranging from 0.78 to 0.79) and well-established construct validity in population-based research [46, 47]. Fourth, our study specifically examined the association between baseline BRI and longitudinal depressive symptom trajectories, rather than the dynamic interplay between evolving BRI and depressive symptoms. This approach aligns with established analytical frameworks investigating how baseline exposures influence subsequent outcome trajectories [3, 48]. Our future work will prospectively explore the dynamic association between longitudinal changes in the BRI and corresponding trajectories of depressive symptoms. Finally, each wave of the CHARLS includes participants of varying ages rather than a single age cohort. At baseline (Wave 1), participants were aged 45 to 93 years, with similar age distributions maintained in subsequent waves. This longitudinal approach enables examination of broad age-related patterns while tracking the same individuals over time. Therefore, consistent with prior researches [3, 35], the observed trajectories in our study reflected development occurring within a specific historical period rather than pure age effects. Future research would benefit from cohort-sequential or accelerated longitudinal designs tracking multiple cohorts across identical age ranges to completely disentangle these factors.

## Conclusion

In conclusion, our longitudinal analysis demonstrates that elevated BRI values are independently associated with more favorable depressive symptom trajectories over time, thereby providing empirical support for the “jolly fat” hypothesis in Chinese middle-aged and older adults. These findings highlight the importance of monitoring psychological status during weight management interventions. Our results provide important insights for early depression detection and prevention strategies in aging populations. Future research should elucidate the biological mechanisms linking BRI to depressive symptoms and evaluate BRI modification as a potential preventive intervention.

## Supplementary Information

Supplementary Material 1

## Data Availability

The data that support the findings of this study are available from the corresponding author upon reasonable request.
