# Supplementary material for: Association between body roundness index and trajectories of depressive symptoms among Chinese middle-aged and older adults: a nationwide cohort study from CHARLS
Source: BMC Psychol. 2025 Nov 18;13:1274. doi: 10.1186/s40359-025-03621-z (PMC12624990; doi:10.1186/s40359-025-03621-z)
Supplement: Supplementary file 1 — Supplementary Material 1 [file 40359_2025_3621_MOESM1_ESM.docx]

**Supplementary Table 1.** Different depressive-symptom trajectory models

| **Group** | **AIC** | **BIC** | **Class proportion (%)** | | | | | | **AvePP** | | | | | |
| --- | --- | --- | --- | --- | --- | --- | --- | --- | --- | --- | --- | --- | --- | --- |
|  |  |  | **Class 1** | **Class 2** | **Class 3** | **Class 4** | **Class 5** | **Class6** | **Class 1** | **Class 2** | **Class 3** | **Class 4** | **Class 5** | **Class 6** |
| 2 | 274,691.50 | 274,800.15 | 51.80 | 48.20 | NA | NA | NA | NA | 0.85 | 0.91 | NA | NA | NA | NA |
| 3 | 274,087.04 | 274,231.91 | 40.19 | 24.89 | 34.92 | NA | NA | NA | 0.83 | 0.86 | 0.69 | NA | NA | NA |
| 4 | 274,040.05 | 274,221.13 | 38.11 | 10.05 | 19.35 | 32.48 | NA | NA | 0.77 | 0.45 | 0.83 | 0.63 | NA | NA |
| 5 | 273,957.57 | 274,174.87 | 38.65 | 20.93 | 21.16 | 11.13 | 8.14 | NA | 0.81 | 0.54 | 0.65 | 0.79 | 0.58 | NA |
| 6 | 273,948.59 | 274,202.11 | 39.74 | 14.25 | 12.93 | 9.47 | 12.22 | 11.39 | 0.79 | 0.45 | 0.52 | 0.58 | 0.77 | 0.47 |

*Note:* NA, Not available; AIC, Akaike Information Criterion; BIC, Bayesian Information Criterion; AvePP, average posterior probability.

**Supplementary Table 2.** Depressive symptom scores alongside corresponding participant ages at each wave

| **Variables** | **2011 wave** | **2013 wave** | **2015 wave** | **2018 wave** | **2020 wave** | ***P*-value** |
| --- | --- | --- | --- | --- | --- | --- |
| Age (year), Mean ± SD | 57.66 ± 8.41 | 59.66 ± 8.41 | 61.66 ± 8.41 | 64.66 ± 8.41 | 66.66 ± 8.41 | < 0.001 |
| CESD-10, Median (IQR) | 7.00 (3.00, 12.00) | 7.00 (4.00, 11.00) | 7.00 (3.00, 12.00) | 8.00 (3.00, 13.00) | 8.00 (4.00, 13.00) | < 0.001 |

*Note:* SD, standard deviation; IQR, interquartile range.

**Supplementary Table 3.** Coefficients for all covariates

| **Variables** | **Model 1** | | **Model 2** | | **Model 3** | | **Model 4** | |
| --- | --- | --- | --- | --- | --- | --- | --- | --- |
|  | **OR (95%CI)** | ***P*-value** | **OR (95%CI)** | ***P*-value** | **OR (95%CI)** | ***P*-value** | **OR (95%CI)** | ***P*-value** |
| BRI | 1.04 (1.01-1.07) | 0.013 | 0.95 (0.93-0.99) | 0.004 | 0.96 (0.93-0.99) | 0.017 | 0.96 (0.93-0.99) | 0.019 |
| Gender |  |  |  |  |  |  |  |  |
| Male | 1 (Ref) |  | 1 (Ref) |  | 1 (Ref) |  | 1 (Ref) |  |
| Female | 2.15 (1.99-2.32) | <0.001 | 2.01 (1.84-2.19) | <0.001 | 2.01 (1.77-2.29) | <0.001 | 2.00 (1.75-2.27) | <0.001 |
| Residence |  |  |  |  |  |  |  |  |
| Rural | 1 (Ref) |  | 1 (Ref) |  | 1 (Ref) |  | 1 (Ref) |  |
| Urban | 0.45 (0.41-0.51) | <0.001 | 0.71 (0.61-0.82) | <0.001 | 0.70 (0.60-0.81) | <0.001 | 0.69 (0.59-0.80) | <0.001 |
| Education level |  |  |  |  |  |  |  |  |
| Elementary school or below | 1 (Ref) |  | 1 (Ref) |  | 1 (Ref) |  | 1 (Ref) |  |
| Middle school | 0.51 (0.46-0.56) | <0.001 | 0.65 (0.59-0.72) | <0.001 | 0.70 (0.62-0.78) | <0.001 | 0.71 (0.64-0.80) | <0.001 |
| High school | 0.33 (0.29-0.39) | <0.001 | 0.50 (0.43-0.58) | <0.001 | 0.55 (0.47-0.65) | <0.001 | 0.56 (0.48-0.67) | <0.001 |
| College or above | 0.13 (0.09-0.21) | <0.001 | 0.30 (0.19-0.48) | <0.001 | 0.36 (0.22-0.59) | <0.001 | 0.37 (0.23-0.61) | <0.001 |
| Marital status |  |  |  |  |  |  |  |  |
| Married | 1 (Ref) |  | 1 (Ref) |  | 1 (Ref) |  | 1 (Ref) |  |
| Not married | 1.72 (1.51-1.96) | <0.001 | 1.47 (1.28-1.69) | <0.001 | 1.55 (1.34-1.80) | <0.001 | 1.58 (1.36-1.83) | <0.001 |
| Age (year) | 1.01 (1.00-1.01) | <0.001 | 1.00 (1.00-1.01) | 0.154 | 1.00 (0.99-1.00) | 0.680 | 1.00 (0.99-1.00) | 0.268 |
| CRP (mg/L) | 1.00 (0.99-1.01) | 0.864 |  |  | 1.00 (0.99-1.00) | 0.197 | 1.00 (0.99-1.00) | 0.177 |
| Health insurance |  |  |  |  |  |  |  |  |
| Urban employee medical insurance | 1 (Ref) |  | 1 (Ref) |  | 1 (Ref) |  | 1 (Ref) |  |
| Urban and rural resident medical insurance | 3.02 (2.60-3.50) | <0.001 | 1.51 (1.23-1.85) | <0.001 | 1.32 (1.07-1.64) | 0.010 | 1.30 (1.05-1.62) | 0.017 |
| Other medical insurance | 1.25 (0.81-1.93) | 0.305 | 0.91 (0.58-1.43) | 0.688 | 0.96 (0.60-1.54) | 0.857 | 0.91 (0.57-1.46) | 0.701 |
| No insurance | 3.21 (2.59-3.99) | <0.001 | 1.77 (1.38-2.26) | <0.001 | 1.70 (1.31-2.20) | <0.001 | 1.71 (1.32-2.22) | <0.001 |
| Household consumption (yuan) | 1.00 (1.00-1.00) | <0.001 | 1.00 (1.00-1.00) | 0.025 | 1.00 (1.00-1.00) | 0.057 | 1.00 (1.00-1.00) | 0.021 |
| Hypertension |  |  |  |  |  |  |  |  |
| No | 1 (Ref) |  |  |  |  |  | 1 (Ref) |  |
| Yes | 1.08 (1.00-1.17) | 0.052 |  |  |  |  | 0.97 (0.88-1.07) | 0.556 |
| Dyslipidemia |  |  |  |  |  |  |  |  |
| No | 1 (Ref) |  |  |  |  |  | 1 (Ref) |  |
| Yes | 1.04 (0.95-1.14) | 0.361 |  |  |  |  | 0.96 (0.86-1.06) | 0.392 |
| Diabetes |  |  |  |  |  |  |  |  |
| No | 1 (Ref) |  |  |  |  |  | 1 (Ref) |  |
| Yes | 1.30 (1.12-1.51) | 0.001 |  |  |  |  | 1.18 (0.99-1.40) | 0.062 |
| Cancer or malignant tumor |  |  |  |  |  |  |  |  |
| No | 1 (Ref) |  |  |  |  |  | 1 (Ref) |  |
| Yes | 1.56 (1.05-2.30) | 0.026 |  |  |  |  | 1.04 (0.67-1.61) | 0.865 |
| Chronic lung diseases |  |  |  |  |  |  |  |  |
| No | 1 (Ref) |  |  |  |  |  | 1 (Ref) |  |
| Yes | 1.85 (1.62-2.11) | <0.001 |  |  |  |  | 1.30 (1.11-1.52) | 0.001 |
| Liver diseases |  |  |  |  |  |  |  |  |
| No | 1 (Ref) |  |  |  |  |  | 1 (Ref) |  |
| Yes | 1.46 (1.19-1.78) | <0.001 |  |  |  |  | 1.04 (0.83-1.31) | 0.712 |
| Heart diseases |  |  |  |  |  |  |  |  |
| No | 1 (Ref) |  |  |  |  |  | 1 (Ref) |  |
| Yes | 1.58 (1.39-1.79) | <0.001 |  |  |  |  | 1.01 (0.87-1.17) | 0.860 |
| Stroke |  |  |  |  |  |  |  |  |
| No | 1 (Ref) |  |  |  |  |  | 1 (Ref) |  |
| Yes | 1.83 (1.35-2.48) | <0.001 |  |  |  |  | 1.34 (0.95-1.89) | 0.090 |
| Kidney diseases |  |  |  |  |  |  |  |  |
| No | 1 (Ref) |  |  |  |  |  | 1 (Ref) |  |
| Yes | 2.07 (1.76-2.44) | <0.001 |  |  |  |  | 1.46 (1.21-1.76) | <0.001 |
| Stomach or other digestive diseases |  |  |  |  |  |  |  |  |
| No | 1 (Ref) |  |  |  |  |  | 1 (Ref) |  |
| Yes | 1.98 (1.81-2.17) | <0.001 |  |  |  |  | 1.30 (1.17-1.45) | <0.001 |
| Memory related diseases |  |  |  |  |  |  |  |  |
| No | 1 (Ref) |  |  |  |  |  | 1 (Ref) |  |
| Yes | 2.01 (1.33-3.04) | 0.001 |  |  |  |  | 1.76 (1.10-2.82) | 0.019 |
| Arthritis or rheumatism |  |  |  |  |  |  |  |  |
| No | 1 (Ref) |  |  |  |  |  | 1 (Ref) |  |
| Yes | 2.28 (2.09-2.47) | <0.001 |  |  |  |  | 1.58 (1.44-1.74) | <0.001 |
| Asthma |  |  |  |  |  |  |  |  |
| No | 1 (Ref) |  |  |  |  |  | 1 (Ref) |  |
| Yes | 1.96 (1.57-2.45) | <0.001 |  |  |  |  | 1.29 (0.99-1.67) | 0.056 |
| Physical activities |  |  |  |  |  |  |  |  |
| Vigorous activities | 1 (Ref) |  |  |  | 1 (Ref) |  | 1 (Ref) |  |
| Moderate activities | 0.88 (0.77-1.02) | 0.085 |  |  | 0.85 (0.73-1.00) | 0.049 | 0.88 (0.75-1.03) | 0.111 |
| Other activities | 0.87 (0.78-0.97) | 0.009 |  |  | 0.85 (0.76-0.96) | 0.007 | 0.89 (0.79-1.00) | 0.045 |
| Social activities |  |  |  |  |  |  |  |  |
| No | 1 (Ref) |  |  |  | 1 (Ref) |  | 1 (Ref) |  |
| Yes | 0.71 (0.65-0.76) | <0.001 |  |  | 0.81 (0.75-0.89) | <0.001 | 0.81 (0.74-0.88) | <0.001 |
| Smoking status |  |  |  |  |  |  |  |  |
| Current smokers | 1 (Ref) |  |  |  | 1 (Ref) |  | 1 (Ref) |  |
| Former smokers | 0.86 (0.74-1.01) | 0.058 |  |  | 0.77 (0.65-0.91) | 0.002 | 0.73 (0.61-0.86) | <0.001 |
| Never smokers | 1.46 (1.34-1.59) | <0.001 |  |  | 0.85 (0.75-0.97) | 0.013 | 0.86 (0.76-0.98) | 0.027 |
| Drinking status |  |  |  |  |  |  |  |  |
| Current drinkers | 1 (Ref) |  |  |  | 1 (Ref) |  | 1 (Ref) |  |
| Former drinkers | 1.23 (1.05-1.45) | 0.009 |  |  | 1.11 (0.93-1.32) | 0.244 | 1.10 (0.92-1.31) | 0.287 |
| Never drinkers | 1.79 (1.63-1.96) | <0.001 |  |  | 1.07 (0.95-1.20) | 0.244 | 1.07 (0.95-1.20) | 0.240 |
| Self-rated health |  |  |  |  |  |  |  |  |
| Good | 1 (Ref) |  |  |  | 1 (Ref) |  | 1 (Ref) |  |
| Fair | 2.10 (1.89-2.33) | <0.001 |  |  | 2.08 (1.87-2.32) | <0.001 | 1.85 (1.66-2.07) | <0.001 |
| Poor | 7.09 (6.28-8.01) | <0.001 |  |  | 6.48 (5.71-7.37) | <0.001 | 5.00 (4.37-5.72) | <0.001 |

*Note*: CI, confidence interval; OR, odds ratio; model 1 was unadjusted; model 2 was adjusted for sociodemographic characteristics; model 3 was further adjusted for behavioral factors and health indicators; and model 4 was additionally adjusted for chronic diseases.
